# Repeated series learning revisited with a novel prediction on the reduced effect of item frequency in dyslexia

**DOI:** 10.1038/s41598-022-16805-z

**Published:** 2022-08-08

**Authors:** Eva Kimel, Itay Lieder, Merav Ahissar

**Affiliations:** 1 The Edmond and Lily Safra Center for Brain Sciences, The Hebrew University of Jerusalem, Jerusalem, Israel; 2 The Department of Psychology, The Hebrew University of Jerusalem, Jerusalem, Israel

**Keywords:** Psychology, Human behaviour

## Abstract

Developmental dyslexia, a difficulty with acquiring fluent reading, has also been characterized by reduced short-term memory (STM) capacity, which is often operationalized with span tasks. The low performance of individuals with dyslexia (IDDs) in such tasks is commonly attributed to poor phonological memory. However, we suggest an alternative explanation based on the observation that many times the items that are used in spans tasks are high-frequency items (e.g., digit words). We suggest that IDDs do not enjoy the benefit of item frequency to the same extent as controls, and thus their performance in span tasks is especially hampered. On the contrary, learning of repeated sequences was shown to be largely independent of item frequency, and therefore this type of learning may be unimpaired in dyslexia. To test both predictions, we used the Hebb-learning paradigm. We found that IDDs’ performance is especially poor compared to controls’ when high-frequency items are used, and that their repeated series learning does not differ from that of controls. Taken together with existing literature, our findings suggest that impaired learning of repeated series is not a core characteristic of dyslexia, and that the reports on reduced STM in dyslexia may to a large extent be explained by reduced benefit of item frequency.

## Introduction

Developmental dyslexia is defined as a specific difficulty in acquiring reading proficiency, manifested in reduced accuracy, fluency, word recognition, decoding, and spelling abilities in spite of hearing, intelligence, and educational opportunities being adequate^1,2^. Dyslexia is usually diagnosed during school years and persists into adulthood^3^. However, dyslexia is manifested not only in difficulties with literacy, and one of its prominent characteristics is poor short-term memory (STM) (e.g.,^4,5^).

STM is typically assessed with the standard Digit Span test (e.g.,^6,7^), and numerous studies report that the performance of individuals with dyslexia (IDDs) in this task is lower than that of controls (e.g.,^8–17^). Performance in span tasks is correlated with reading speed within and across languages^18,19^, suggesting functional relations between poor span scores and poor reading skills in the case of dyslexia^4,20–22^.

Overall, the observation of poor scores in span tasks among IDDs, including when non-words are used (e.g.,^4,20–22^), is very robust (though see^23^). This impaired performance has been traditionally attributed to poor long-term phonological representations (e.g.,^6^) or to reduced efficiency in accessing these representations when performing an STM task^9,24^. However, the hypothesis that the core deficit in dyslexia is poor phonological representations has been challenged by many studies in the past two decades^25^. First, studies showed that IDDs suffer from additional deficits and second, IDDs demonstrate normal performance in some tasks that require adequate phonological representations^26^. Furthermore, dyslexia probably has more than one single cause^27,28^.

The focus of this study is the reduced benefit of input statistics in dyslexia. The *anchoring deficit hypothesis*^29–31^ pertains to IDDs’ learning dynamics, and proposes that their learning process does not produce the same benefit from input statistics as it does for controls^12,32,33^, leading to impoverished cortical representations of highly frequent categories^34,35^.

Span tasks provide an informative window into the use of long-term statistics, since repeated long-term exposure has a large effect on the general population’s performance in these tasks. For the general population, spans of frequent words are larger than those of infrequent ones^36,37^; the span of words is larger than the span of non-words^38^; repetition accuracy of a multi-syllabic non-word is higher when the frequency of the first syllable is high^39^; and recall of sequences of syllables is higher for syllables that occur frequently in polysyllabic English words than it is for less frequent ones^40^.

With the foregoing in mind, we reasoned that if IDDs’ rate of accumulation of input statistics is reduced, their relative difficulties will increase with exposure compared to their peers without dyslexia who received similar exposure. This prediction relies on a well-documented observation in the literature of skill acquisition and learning: although the rate of learning decreases with exposure (i.e., “diminishing returns”), improvement continues over hundreds of thousands of exposures, practically infinitely. This non-saturating characteristic of learning (the exponential/power law of practice^41,42^) has been shown across domains, including language statistics^43^. If this is the case, we expect that the benefit of frequent items will be smaller among IDDs. However, if IDDs deficit is fixed (i.e., it is not a function of exposure and learning dynamics), IDDs’ relative performance is not expected to be especially deteriorated for frequent items.

**Figure 1:**
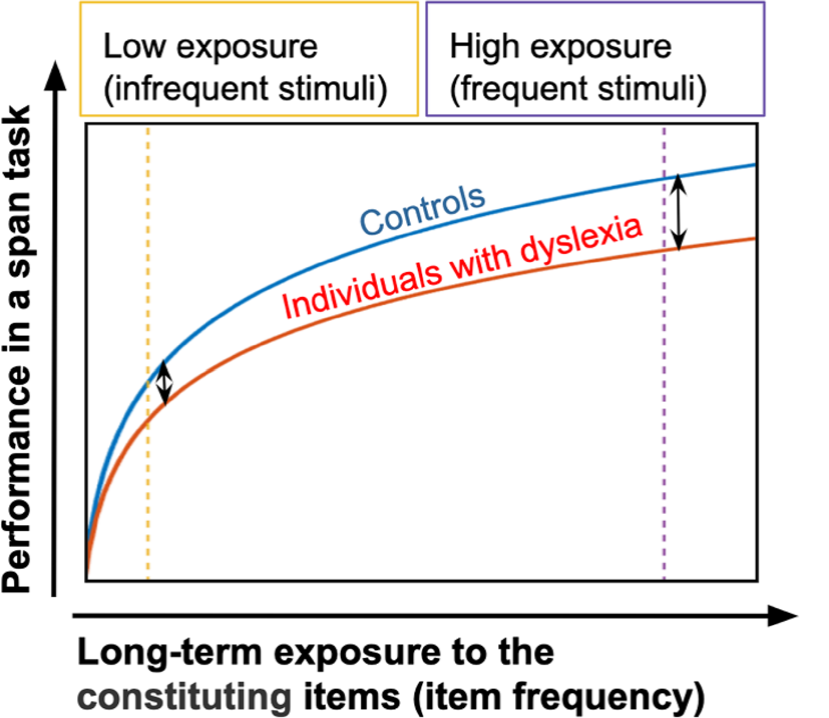
An illustration of the predicted performance in a span task as a function of exposure to the items that are used in the span task. Since the rate of increase is shallower for individuals with dyslexia (slower learning), group difference is expected to increase with exposure. Therefore, group difference is expected to be larger for frequent (right dashed line) than for infrequent (left dashed line) stimuli. Figure adapted from^13^.

Specifically, this should be the case for item frequency in span tasks. Repeated exposure to the items (i.e., greater frequency) was shown to increase performance in span tasks in the general population, and we suggest that this benefit from item frequency follows a learning curve that satisfies the following constraints: (1) the benefits become smaller with exposure, (2) learning does not saturate. This hypothesis is illustrated in Fig. 1 (blue curve).

Assuming that IDDs’ learning curve follows the same shape of controls’ learning curve but with smaller improvement, their proficiency is not expected to catch up because step size decreases with exposure, the learning function is not saturating, and thus group difference will only increase with practice. Consequently, both controls and IDDs will improve given more and more exposure to the items that constitute the spans, but the advantage afforded by each exposure will be larger for controls, and hence group difference will increase with exposure leading to a greater group difference in the frequent, larger exposure, condition (right arrow in Fig. 1). Technically speaking, this predicts a *Group* × *Stimulus Frequency* interaction when the difference in stimulus frequency is sufficiently large.

Importantly, according to all theories focused on learning rather than on reduced skills as a fixed characteristic of dyslexia^44,45^, a group difference that increases with practice is theoretically predicted, given that there is no absolute maximum level of performance. One prediction derived from such learning-based approaches is that IDDs might also underuse linguistic structural regularities that are implicitly acquired over time. Indeed, it had been previously reported that when compared with the general population, IDDs rely less on repeated word structure—morphology, when acquiring new vocabulary and during reading^12^; that they have reduced sensitivity to morphological similarity between words^46^; and that they show slower and less accurate production of noun and adjective inflections^47^ as well as impaired morphological relationship judgments^48^.

To summarize, item frequency effect in span tasks provides an opportunity to test theories of the mechanisms underlying dyslexia: According to theories that view IDDs' phonological deficits as a cause rather than an accumulative outcome of learning deficits, less frequent phonological items are predicted to pose greater difficulties to IDDs than frequent stimuli, since massive exposure provides an opportunity to (at least partially) compensate for the phonological deficit. However, we propose that IDDs do not benefit from item frequency to the same extent that controls do, and that their relative difficulties will therefore increase with item frequency. Thus, although exposure will facilitate performance of all individuals, it will have a smaller effect on that of IDDs^13^. Hence, the group effect will increase with item frequency, in accordance with theories that focus on slower learning rate (as illustrated in Fig. 1).

Following this line of thought, Kimel et al.^13^ have administered a series of span experiments, using frequent and infrequent syllables. Indeed, they found that IDDs benefit less than controls from syllable frequency, and that group difference is larger for frequent items than for rare items. Kimel et al.^13^ have used a paradigm where the length of the sequence to be remembered is gradually increasing like in the standard Digit Span paradigm. While this is a valid and frequently used design, it might encourage the utilization of additional strategies based on the gradual increase of sequence length. We therefore decided to use a paradigm which controls for this potential confound: the Hebb-repetition learning paradigm^49^, in which all sequences have the same length.

In addition to the strong effect of single-item frequency, performance in span tasks is greatly affected by sequence repetition. Kimel et al.^13^ also addressed the benefit from sequence repetition among four different populations: IDDs, controls, musicians, and non-native speakers. They found that controls and IDDs did not differ in their benefit from sequence repetition. However, the task that they used introduced a relatively small number of sequence repetitions, and these allowed gradual learning as explained above. Using the Hebb-learning paradigm^49^, in which the same repeated sequence is presented many times and which was specifically designed for assessing the learning of repeated sequences will also deal with this potential confound.

The Hebb-learning task is an elegant paradigm that is specifically designed to assess sensitivity to repetition of the serial order^49^. In this design, all sequences comprise the same items in a different order, and one specific sequence is repeated every third trial. Participants’ recall of the repeated sequence improves significantly compared with their recall of the non-repeated sequences, indicating specific benefits from sequence repetition (*The Hebb repetition effect*^49^). Since all sequences are composed of the same items, this paradigm allows to specifically track the rate of learning for a repeated sequence (though note^50,51^). Importantly, the number of sequences administered (30) is larger than in Digit Span, does not depend on participants’ performance, and the scoring is per item rather than per sequence, thus providing more information.

The Hebb repetition-learning paradigm has been administered to IDDs in previous studies with mixed results. Two studies reported reduced benefits from serial repetition of sequences of Consonant–Vowel syllables presented auditorily, visually, and for spatial configurations of dots^52,53^. However, a follow-up replication study^54^ did not reproduce this effect:  They administered the same tasks to both children and adults with and without dyslexia, and found no deficit in IDDs’ Hebb repetition effect: neither between the two groups of children nor between the two groups of adults. Similar results were found in additional studies^54–56^. Finally, when adult IDDs participated in a Hebb-learning paradigm with short non-words, they showed a tendency to reduced serial learning, though the effect was not significant^14^. To summarize, the majority of studies report no deficiency among IDDs, or only a mild deficiency, in the task of repeated sequence learning.

Based on this, and on the relatively small number of repetitions in the Hebb-learning paradigm (10 repetitions; at the very left in Fig. 1), we predicted that IDDs’ difficulties will not be with learning a repeated sequence. But, that IDDs will have special difficulty with spans when frequent items are the ones composing the sequences (right hand side in Fig. 1). To test this, we administered the Hebb-learning paradigm twice: once with frequent (Consonant–Vowel, CV) syllables and once with rare (Vowel-Consonant, VC) syllables. This allowed us to assess the impact of syllable frequency on series recall in general, and the impact of syllable type on the learning of repeated series. We expected a smaller group difference for the less frequent syllables (left arrow in Fig. 1), and a bigger difference for the frequent syllables (right arrow in Fig. 1). Importantly, the phonological load is equal or smaller in the frequent (CV) condition as these syllables are easier to perceive and pronounce^57,58^. But, since we think that the core deficit in dyslexia is inefficient learning and not a static phonological deficit, we predict that group difference will be larger in this phonologically-easier condition. This is due to the special benefit of frequency that this condition allows to controls but less so to IDDs.

The double application of the Hebb-repetition protocol, with frequent and rare items, also allowed us to dissociate between long-term item frequency and sequence learning, often confounded in studies of statistical learning in language^59^. A theoretical dissociation between sensitivity to conditional and sensitivity to distributional statistical structure was previously suggested^60^. Moreover, brain studies suggest an anatomical dissociation between the mechanisms underlying sequence learning and those underlying sensitivity to distributional statistics, the latter of which underlies the representation of categories and prototypes^34^. To wit, evidence from imaging and patients suggests that the learning of a repeated sequence relies on the hippocampus^61–63^ and that this learning is impaired when hippocampal structures are impaired^64^, whereas categories are represented semantically^65^ and perceptually^66,67^ in specific cortical regions. To the best of our knowledge, there are no reports of abnormal hippocampal activity in dyslexia that is related to sequence learning^63^. Hence, based on brain studies, there is no reason to assume impaired sequence learning in dyslexia^52,53^.

We focused on syllables as the basic items for the Hebb-repetition task since exposure to them does not strongly depend on reading experience, and hence the exposure of controls and IDDs is expected to be similar. Syllables’ presence as separate mental entities is evident quite early in development, whereas phonological awareness at the phonemic level requires explicit instruction and is reciprocally related to literacy (e.g.,^68^). Indeed, illiterate adults and nursery-school children show similar rates of success at manipulating syllables, but only literate individuals successfully operate with phonemes^69,70^. Syllables’ unique psychological reality is retained in reading, where syllables probably form the basic automatically extracted unit^71–73^.

To summarize, we predicted a reduced benefit from syllable frequency for IDDs due to a reduced benefit from each exposure, as described in Fig. 1. With regard to repeated sequence learning, we did not expect to find a group difference, based on brain activity studies (e.g.,^62,74^), and on previous behavioral research^13,14,54–56^ (though see^31,52,53^).

## Results

The results of the Hebb repetition paradigm were consistent with our predictions: IDDs showed adequate benefits from series repetition, indicating adequate serial-order learning, both when assessed with the slope and with mean performance. At the same time, IDDs’ scores were especially low compared to controls’ when high-frequency syllables were used.

### Analysis of mean scores

**Figure 2:**
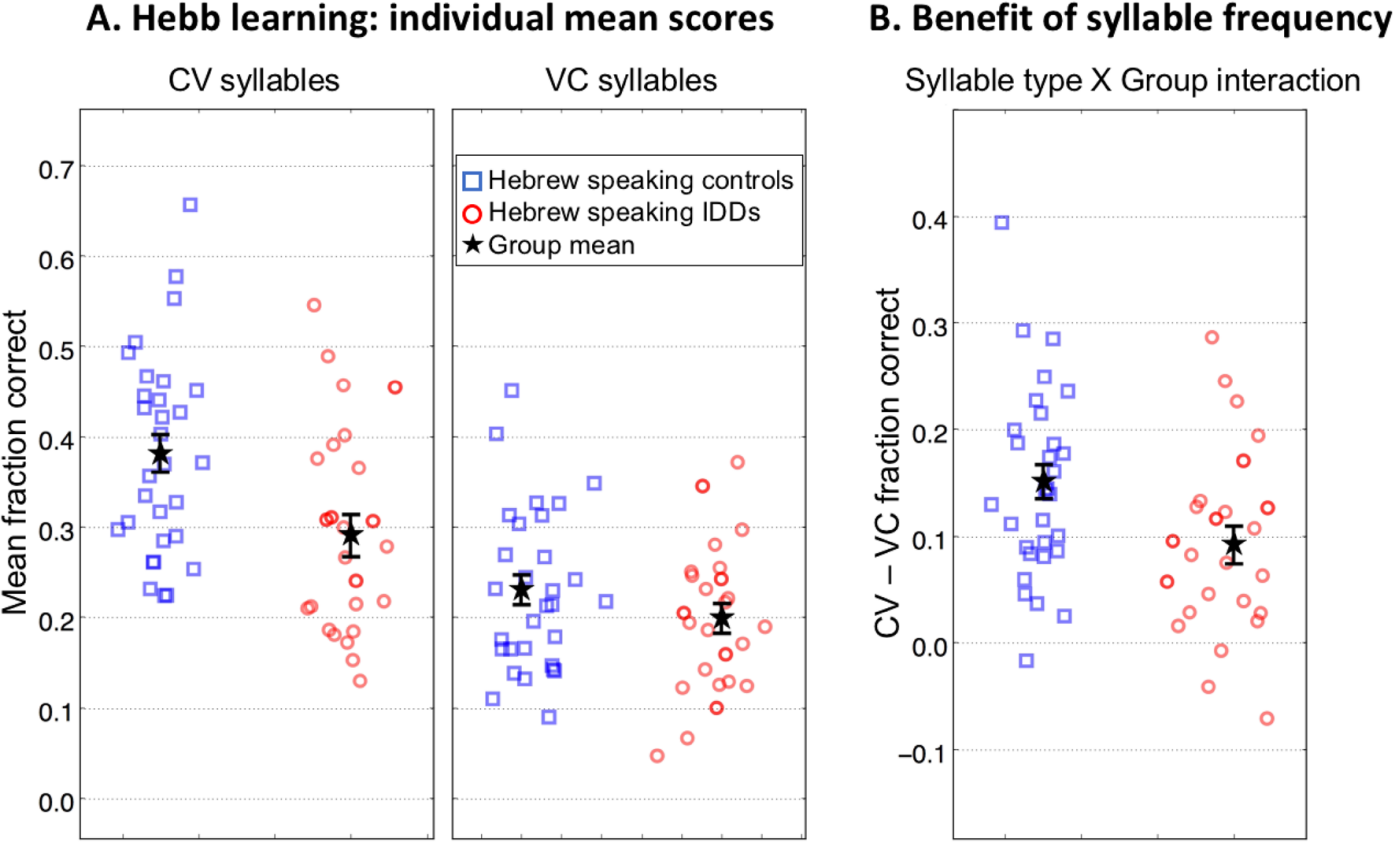
Hebb repetition-learning paradigm: sensitivity to order using frequent (CV) and infrequent (VC) syllables: Hebrew speakers, controls vs. IDDs. (**A**) Mean scores across all sequences in the Hebb-learning protocol of controls (blue squares) and of IDDs (red circles). Left: frequent (CV) syllables. Right: infrequent (VC) syllables. (**B**) Benefits in spans (score differences) of CV compared with VC (long-term frequency effect). Controls gained more than IDDs from the use of the frequent CV syllables. Each symbol denotes a single participant's mean score across all 30 sequences of the Hebb-learning protocol. Error bars denote one SEM.

**Figure 3:**
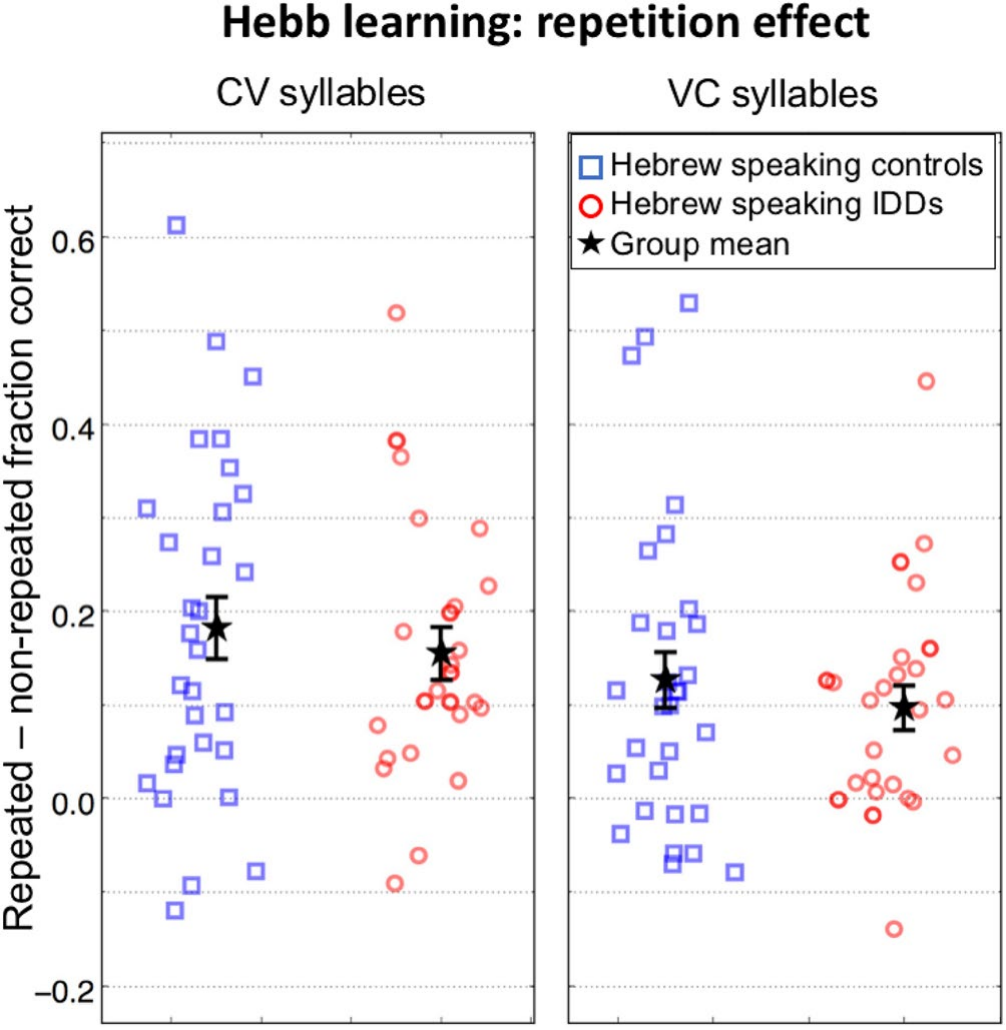
Difference between scores for repeated (Hebb) vs. non-repeated (filler) sequences of CV (left) and VC (right) syllables. The benefit is larger for CV syllables and is similar for controls (blue squares) and IDDs (red circles). Symbols denote individual difference scores. Error bars denote one SEM. The values are slightly jittered for display purposes.

We used a mixed-design ANOVA with *Syllable Type* (frequent vs. infrequent) and *Repetition* (repeated vs. non-repeated sequences) as within-participant factors, and *Group* (controls vs. IDDs) as a between-participant factor. The expected main effects were attained: scores of frequent syllables were higher than scores of infrequent syllables (main effect of *Syllable Type*: *F*_(1,53)_ = 86.47, *p* < 0.001, *η2* = 0.620; Fig. 2; Table 1); scores of the repeated sequence were higher than scores of the non-repeated sequences (main effect of *Repetition*: *F*_(1,53)_ = 63.99, *p* = 10^–9^, *η*^2^ = 0.547; Fig. 3; Table 1); controls had higher scores than IDDs (main effect of *Group*: *F*_(1,53)_ = 5.43, *p* = 0.024, *η*^2^ = 0.093; Fig. 2A; Table 1).

**Table 1:** Hebb learning: mean scores and slopes.

Type of syllable	Type of sequence	Scores		Slopes	
		Controls	IDDs	Controls	IDDs
Frequent	Non-repeated	0.32 (0.07)	0.24 (0.10)	0.005 (0.02)	− 0.00 (0.01)
	Repeated	0.50 (0.20)	0.39 (0.18)	0.030 (0.03)	0.023 (0.02)
	Average repeated and non-repeated	0.41	0.31	0.017	0.011
Infrequent	Non-repeated	0.19 (0.06)	0.17 (0.07)	0.004 (0.01)	− 0.003 (0.01)
	Repeated	0.31 (0.18)	0.26 (0.14)	0.017 (0.03)	0.011 (0.03)
	Average repeated and non-repeated	0.25	0.21	0.010	0.004
Overall average		0.33	0.26	0.014	0.008

Scores (mean (SD)) and slopes (mean (SD)) of Hebb repetition-learning with frequent and rare syllables. The score for each sequence was between zero and one: zero if none of the nine syllables composing the sequence was recalled correctly with respect to both its identity and serial position, and ~ 0.11 for each correctly recalled syllable. The total score for each participant for each condition is the average of the sequences’ scores. The slopes of the regression lines were calculated for each individual in each condition based on performance as a function of trial number.

Both groups significantly benefited from syllable frequency (controls: *mean difference* = 0.160, *SE* = 0.02, *p* = 10^–10^; IDDs: *mean difference* = 0.101, *SE* = 0.02, *p* = 10^–5^). However, importantly, controls benefited more than did IDDs (*Syllable Type* × *Group*: *F*_(1,53)_ = 4.37, *p* = 0.041, *η2* = 0.076; Table 1; Fig. 2B). In order to verify that the scores of IDDs for the infrequent syllables are not “at floor”, we have checked the skewness and kurtosis of the data of IDDs in the infrequent syllable condition: *skewness* = 0.369, *kurtosis* = -−0.31. These values indicate that this is not likely to be a floor effect^75^.

As expected, there was no significant group difference in the repetition effect (*Repetition* × *Group*: *F*_(1,53)_ = 0.64, *p* = 0.427, *η*^2^ = 0.012; Table 1; Fig. 3). The lack of difference between the groups (“null” hypothesis) was supported (though not strongly) by the value of the Bayes factor (B_01_): Scaled JZS Bayes Factor = 3.7, calculated on the benefit from repetition^76^.

Though repetition effect was significant for both the frequent CV syllables (*mean difference* = 0.168, *SE* = 0.02, *p* < 10^–9^; Fig. 3—left) and the infrequent VC syllables (*mean difference* = 0.111, *SE* = 0.02, *p* = 10^–6^; Fig. 3—right), the benefit from repetition was larger for frequent (CV) than for infrequent (VC) syllables (*Repetition* × *Syllable Type*: *F*_(1,53)_ = 6.01, *p* = 0.018, *η*^2^ = 0.102; Fig. 3). There was no significant group difference in this repetition benefit for frequent syllables as compared with that of infrequent ones (*Repetition* × *Syllable Type* × *Group*: *F*_(1,53)_ = 0.003, *p* = 0.958, *η*^2^ = 10^–4^; Fig. 3).

To test the connection between benefit from item frequency and performance in STM tasks among IDDs further, we tested the correlation between the benefit from item frequency in the Hebb task, and Digit Span forward score in the dyslexia group. The benefit of item frequency was calculated by subtracting the score for non-repeated infrequent-VC syllables from the score for non-repeated frequent-CV syllables. We did not find a significant correlation (*Spearman’s ρ* = 0.27, *p* = 0.187).

We also tested the association of the benefit of frequency with the main defining symptom of dyslexia: poor reading. The benefit of item frequency was calculated by subtracting the score for non-repeated infrequent-VC syllables from the score for non-repeated frequent-CV syllables. We found a significant correlation with non-word reading accuracy in the dyslexia group: *Spearman’s ρ* = 0.53, *p* = 0.007; but the correlation with word reading accuracy was not significant: *Spearman’s ρ* = 0.34, *p* = 0.094.

### Analysis of learning rates

The Hebb-learning paradigm^49^ also allows for an assessment of learning rates by comparing slopes of the regression lines based on performance in repeated and non-repeated sequences as a function of trial number for each individual^52^. This analysis ignores the baseline performance, and incorporates only the learning rate. We calculated a regression line for each condition for each individual; the standard errors of the estimated gradients were small, and did not exceed 0.005 for any regression line, suggesting that the quality of the fit was satisfactory.

**Figure 4:**
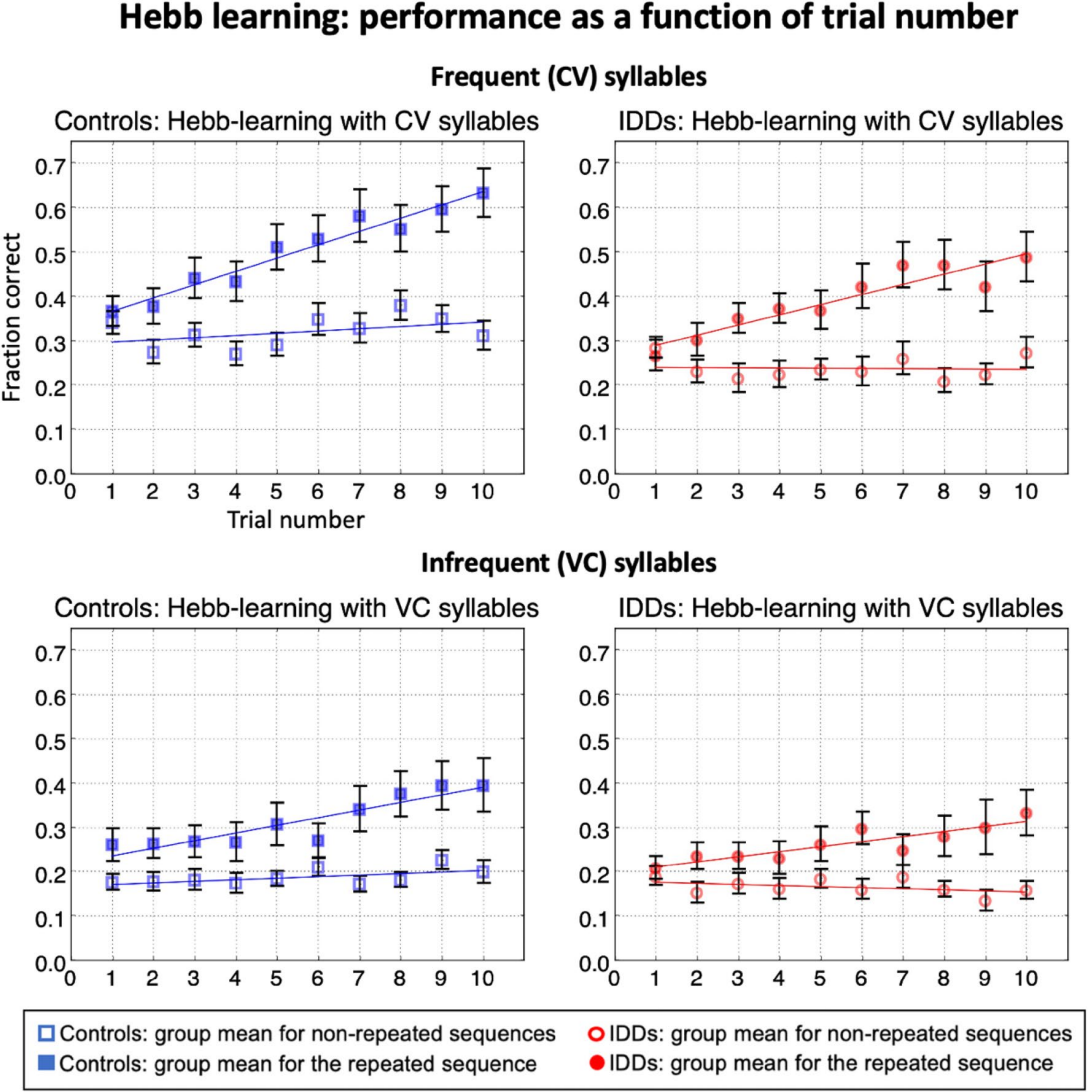
Fraction correct as a function of trial number for both repeated and non-repeated sequences of controls (left) and IDDs (right), for CV (top) and VC (bottom) syllables. Though controls' CV scores for the non-repeated sequences are higher than IDDs' (the regression line on the left has a higher intercept than the one on the right), the repetition-learning slopes do not differ between the groups. Error bars denote one SEM.

Learning rates for frequent syllables were higher than those for infrequent syllables (main effect of *Syllable Type*: *F*_(1,53)_ = 8.62, *p* < 0.005, *η*^2^ = 0.140; Fig. 4—top vs. bottom; Table 1), with no significant group difference (*Group* × *Syllable Type*: *F*_(1,53)_ = 0.01, *p* = 0.942, *η2* = 10^–4^; Fig. 4—left vs. right; Table 1). Learning rates for repeated sequences were higher than those for non-repeated ones, as predicted (main effect of *Repetition*: *F*_(1,53)_ = 36.21, *p* = 10^–6^, *η*^2^ = *0.4*06; Table 1). Importantly, sequence repetition produced no significant group difference with respect to the learning rate (*Group* × *Repetition*: *F*_(1,53)_ = 0.01, *p* = 0.913, *η*^2^ < 0.001). The lack of difference between the groups (“null” hypothesis) is supported (though not strongly) by the value of the Bayes factor (B_01_): Scaled JZS Bayes Factor = 4.9, calculated on the benefit from repetition^76^. Repetition benefited the learning rate of frequent syllables more than it did infrequent ones (Fig. 4; *Repetition* × *Syllable Type*: *F*_(1,53)_ = 4.27, *p* = 0.044, *η*^2^ = *0.0*75; frequent: *mean difference* = 0.024, *SE* = 0.004, *p* = 10^–7^, infrequent: *mean difference* = 0.014, *SE* = 0.004, *p* = 0.002), showing no significant group difference in the repetition benefit to learning rate for frequent vs. infrequent syllables (Fig. 4*; Repetition* × *Group* × *Syllable Type*: *F*_(1,53)_ = 0.03, *p* = 0.865, *η2* < 0.001). In general, there was no significant group difference in the overall *learning rate* throughout the experiment (Fig. 4, main effect of *Group F*_(1,53)_ = 2.73, *p* = 0.104, *η2* = 0.049; Table 1)*.*

The last trials of the experiment might seem to demonstrate stronger learning in controls vs. IDDs. To test this, we have analyzed each of the last 4 learning trials separately, and there was no significant group difference in any of them, strengthening our conclusion of no group difference (maximal *t* = 1.58, *p* = 0.12).

### Analysis of the Hebb-learning paradigm reliability for assessing repeated sequence learning

We have tested the stability of the two measures of repeated series learning: mean scores and learning rates (slopes) for repeated sequences. To do so, we have calculated the correlation between the CV and VC parts of the experiment (two separate runs, similarly to the method used in^51^). The correlations were tested separately for each group, and each of the two scores: Means—controls: *Spearman’s ρ* = 0.656; *p* = 10^–4^, IDDs: *ρ* = 0.46; *p* = 0.022; Slopes—controls*: ρ* = 0.28, *p* = 0.141, IDDs: *ρ* = 0.49; *p* = 0.014). After a Bonferroni correction, 3 out of the 4 correlations came out statistically significant. The lack of correlation of the slopes for the control group could stem from heterogeneity in the benefit of syllable type. Namely, some individuals in the control group might have especially large benefit from performing the task with CV vs. VC syllables, thus hampering the correlation. This is also consistent with a recent study which assessed the reliability of the Hebb-learning task and arrived at the conclusion that only the mean is a stable measure for individual performance^51^. As the main results of the study were replicated for both the mean and the slope measures, we think that the findings of the current study can be used and interpreted.

### Summary of results

Both for *mean performance* and for *learning rate* we found: (1) a significant benefit of sequence repetition, (2) a significant benefit of syllable frequency, (3) a significant *Repetition* × *Syllable Type* interaction: Repetition was more beneficial for CV than for VC syllables, (4) no significant *Repetition* × *Group interaction:* Benefits from repetition did not significantly differ between the groups.

Overall, the dyslexia and control groups demonstrated similar *rates of learning* of the serial order (Figs. 3, 4). On the contrary, when *means* were analyzed, there was a significant group difference in performance, and a *Syllable Type X Group* interaction: controls’ benefit of syllable frequency was higher than that of IDDs (Fig. 2).

## Discussion

We used Hebb repetition-learning, designed for assessing the learning of repeated serial order^49^. We found that controls benefited more than IDDs from the long-term syllable frequency, yet the two groups did not significantly differ in their benefits from within-session sequence repetition.

The dissociation between the impact of item frequency and the impact of sequence repetition on IDDs suggests that sensitivity to single-item long-term distribution and sensitivity to repeated sequences might reflect separate mechanisms, and only the former is impaired in dyslexia. These observations are in line with recent findings by Kimel et al.^13^, which demonstrated that individuals with dyslexia have particularly poor spans compared with controls for frequent syllables in their native language, but an adequate benefit from partial sequence repetition.

Reduced utilization of item frequency is expected to impede reading proficiency, which heavily relies on benefits from familiarity with syllables (e.g.,^73^), word-structure (e.g.,^12,43,46^), and words (e.g.,^78^). However^12,43,46,73^, in what seems to be a contradiction to IDDs’ special difficulty in the frequent condition, IDDs’ deficit in non-word reading is larger than in word reading^79^. We suggest that this stems from an adequate utilization of semantics by IDD. We did not test this question directly, but the results of the current study support this suggestion since while we found a significant correlation between the benefit of syllable frequency and decoding in reading (i.e., non-word reading accuracy), we found weaker, non-significant, correlations when semantics could be used to boost performance (i.e., correlations between the benefit of syllable frequency and word reading accuracy, and between the benefit of syllable frequency and Digit Span score).

### Dyslexia is associated with atypical learning dynamics

Our findings that IDDs benefit less from the use of high-frequency items are consistent with the anchoring deficit hypothesis^30^ and with other theories and studies of dyslexia that emphasize the atypicality of the learning process in this population^80–82^ as illustrated in Fig. 1. The reduced contribution of exposure to processing and to further learning among IDDs was previously demonstrated with pure tones^29,32,33,83^, language sounds^34,82,84–86^, syllables^13^, and words^12,47,87,88^.

From a perceptual perspective, temporal and sequential processes are engaged in reading acquisition. And indeed, deficits with auditory frequency discrimination were numerously reported in studies with IDDs (^32,89–94^, though see^95,96^). As these are low-level processes, a reasonable suggestion was put forward—that is, that the core deficit in dyslexia is poor rapid temporal processing (e.g.,^10,97–99^). However, succeeding studies questioned this claim^29,30,100–103^. For example, Share and colleagues^103^ followed 453 children from kindergarten to grade 2 and found that early temporal deficits did not predict dyslexia. Furthermore, a recent meta-analysis confirmed that auditory perception is worse among IDDs; however, the explanatory power of this low-level deficit for phonological awareness and reading is low or task-specific^104^. Thus, it seems that it is not low-level temporal or sequential perception per-se that is impaired in dyslexia, but rather the utilization of stimuli statistics is less efficient^29–31,34,83,105^, and this reduced efficiency might be domain-general and processing-level independent^106^. We suggest that this is the mechanism that is responsible for the deficiency of IDDs also with language processing^101^ (as depicted in Fig. 1).

A subtle deficit might also exist in short-term learning (i.e., low exposure condition; left-hand side of Fig. 1), although long-term accumulation results in a stronger effect. For example, in a study with language sounds, short-term exposure to language sound-distribution did not assist young IDDs in forming phonemic representations to the same extent as it assisted controls^84^. Similar results were obtained when IDDs and controls were exposed to pure tones^29,32,33,105^, and to words^30^.

Unlike theories of dyslexia in which the learning process is central and is used to account for failed skill acquisition, the phonological deficit account^107^ and external or internal general noise accounts^108,109^ are less likely to explain the results of this study. More exposures allow for more learning opportunities, potentially reducing the effect of noise on performance with the frequent stimuli (or, at least, not increasing it).

Thus, if we assume that the core deficit in dyslexia stems from a static phonological deficit or increased noise, we would expect to find a special difficulty for IDDs in the infrequent condition, contrary to the results of the current study (see also^13^). Indeed, the link between noise exclusion and reading abilities in dyslexia was shown to be weak^110^. Hence, theories that do not take into account the atypicality of the learning process, predict that group difference should not increase with exposure.

### Concatenation as a supporting mechanism for sequence learning

The results suggest that sequence learning in the course of repeated presentations does not strongly depend on single-item long-term frequency since IDDs showed an impaired benefit of item frequency but an adequate benefit of sequence repetition. However, in both groups, the benefits from sequence repetition were larger for the frequent CV than for the infrequent VC sequences. Why should that be the case if sensitivity to repeated serial order does not heavily depend on item frequency? We attribute this disparity to an exposure-independent (i.e., not frequency) difference between CV and VC syllables—a difference in the manner of their articulation that causes CV sequences to be more physically and physiologically “chunkable”. Based on their different types of ending (rime), CV syllables are categorized as "light" whereas VC syllables are categorized as “heavy”^111,112^. Heavy syllables attract stress (the Weight-Stress Principles^112,113^) and a word can only have one primary stress. Therefore, a sequence of VC syllables tends to be articulated with a stress on each syllable, supporting segmentation rather than concatenating, and perhaps impeding memory for the sequence as a whole, since stressed syllables are treated as word onsets^114^. By contrast, a sequence of CV syllables can be perceived and articulated as a long (chunked) multisyllabic word.

The observation that IDDs’ benefits from sequence repetition do not differ from controls’ suggests that the two groups have similar benefits from the manner that CV and VC syllables are produced. Thus, we propose that both groups benefit more from sequence repetition of CV syllables vs. VC syllables because CV syllables tend to be concatenated, and thus chunked, whereas VC syllables do not. Overall this pattern of results suggests that using items with high frequency enhances overall performance, but it does not specifically enhance the benefit of sequence repetition^13^.

One might ask whether these exposure-independent differences can also explain the reduced performance of IDDs in non-repeated sequences composed of CV syllables vs. VC syllables. We have previously assessed this question experimentally and showed that this is not the case^13^. Specifically, a group of native English speakers without dyslexia performed a CV span task. CV syllables are less frequent in English than in Hebrew, and indeed, the performance of this group was significantly lower than that of native Hebrew speakers. However, their benefit of sequence repetition was similar to that of native Hebrew speakers. This suggests that while the absolute performance in span tasks heavily depends on item frequency, repeated sequence learning relies on exposure-independent features.

### Repeated sequence learning in dyslexia: at most a mild deficit

We found adequate sequence learning in dyslexia. This observation is in contrast to earlier reports of poor serial learning in dyslexia in the Hebb-repetition task^52,53^ and of reduced benefit from repeated sequences in other STM tasks^31^. However, it is consistent with a number of other studies^13,14,54–56^ that found no significant deficiency in IDDs’ memory for repeated series. Overall, 6 studies (5 research groups) did not find a significant deficit in IDDs’ repeated-order learning, while 3 studies (2 research groups) reported a significant deficit^13,14,31,52–56^. The 3 studies that report deficit in repeated series learning among IDDs might be reporting a less-typical result^54^ since sample sizes were relatively small in two of the studies, and the effects were marginal^31,52,53^ (Oganian et al.: F(1, 54) = 5.51, p < 0.05; Bogaerts et al.: F(1,46) = 4.73, p < 0.05, F(1,34) = 5.52, p < 0.05, Szmalec et al.: F(1, 30) = 23.22, p < 0.001—three experiments in 3 different modalities were pooled together, but the effect for each of them separately is much weaker). Taken together with the fact that 5 research groups reported a null effect, we suggest that the participants for which a significant deficit was reported might have been less typical^54^. This suggestion is further supported by an atypical finding in the study by Szmalec and colleagues^52^: there was no Hebb-learning in the dyslexia group in one of the experiments.

Another probable contribution to these mixed observations is the low test–retest reliability of the individual learning rate of the repeated sequence, which might lead to unclear results^51^. With regard to this study, we were able to assess the reliability of the measures which were used for assessing repeated series learning as we had two separate Hebb experiments, for CV syllables and for VC syllables. Thus, similarly to Bogaerts and colleagues^51^, we calculated the correlation between the two runs to verify that it is significant, and when we discovered that it was (for 3 out of 4 correlations), we could continue with the interpretation of the results. To further strengthen the reliability of our results, we used both the mean scores and the slopes.

The observation of IDDs’ adequate sequence learning might seem at odds with studies assessing the ability of IDDs to remember the order of items, whose identity they need not remember^115–118^. Memory for order and memory for items in STM tasks were shown to be separate (e.g.,^119^), and a specific deficiency in memory for order was reported for IDDs (^120,121^, though see^122^). For example, Perez et al.^123^ presented a series of animal names and then asked participants to order cards with pictures of these animals. Children with dyslexia performed poorer than both age-matched controls and reading-level-matched children.

However, in spite of the seeming similarity of the assessed cognitive ability, the rate of learning a repeated series (items + order) may rely on a separate mechanism than that which underlies the recall of the order (only) of a non-repeated series. As suggested by the studies described above, memory for order might be impaired in dyslexia and since we have not administered a separate task which requires only memory for order, we cannot exclude the possibility that poor memory for order has contributed to poorer mean performance of IDDs, but in a way that is not specific to the repeated sequence. It would be interesting to add a separate task which measures memory for order to directly test the relations between these types of memory, as these three lines of research: benefit from item long-term frequency, memory for order, and benefit from series repetition, are complementary.

Similarly, it is difficult to compare the skills needed for Hebb learning to the skills required for learning repeated sequences while extracting them from a continuous stream. For example, Gabay et al.^17^ reported a difficulty among IDDs in a statistical learning task that involves segmentation of a continuous stream. They used a stream of syllables and a stream of tones, and for both stimulus types they found that IDDs were less efficient than controls in the extraction of the transitional probability structure. However, van Witteloostuijn and colleagues^124^, who conducted an experiment with a similar design using visual stimuli, did not find a group difference. Moreover, they used two additional paradigms that rely on statistical regularities: the Serial Reaction Time task and an auditory non-adjacent dependency learning, and no group difference emerged for these as well^124^. This emphasizes the notion that learning in such tasks depends on a broad range of skills that are not fully understood^14,50^. In this context, our study makes an important contribution since it dissociates the benefit of long-term item frequency (which IDDs do not utilize as efficiently as controls) from the benefit of within-session series repetition (e.g., Hebb learning; for which IDDs do not differ from controls).

### The neural basis of sequence vs. item learning in dyslexia

A dissociation between the learning of sequences and of singe items, as evident in the results, also emerges from brain studies: while learning of repeated series (i.e., of two and more items) is associated with the hippocampus, object familiarity, modified according to distributional statistics, is represented in the neocortex according to its perceptual categorization.

Specifically, the hippocampus was shown to be engaged during an experiment with the Hebb-learning paradigm^62^, and with other sequence learning tasks^63^. Beyond correlation, causality was also shown: an individual with a hippocampal lesion had specific difficulties in series learning^64^ (though see^125^). By contrast, cortical areas are associated with the storage and access to individual items and categories^65–67^.

To the best of our knowledge, there are no reports of abnormal hippocampal activity in dyslexia, in line with our observation of adequate sequence learning. By contrast, abnormal dynamics of perceptual retention in dyslexia is observed throughout the cortex (e.g.,^74,126^), in line with our observation of reduced benefit from item frequency. Importantly, massive exposure to repeating sequences might eventually result in a significant group difference since after many repetitions sequences may become individually represented items and then “migrate” to the cortex^127^. Namely, fast initial learning relies on the hippocampus, and subsequent consolidation in the superior temporal region, which involves inferior frontal and premotor regions.

Indeed, there is evidence on special difficulties of IDDs with learning a repeated series of key presses, when learning with more than a single session of the Serial Reaction Time task^128^. The brain regions involved in motor sequence learning are broad and include the cerebellum, the striatum, and some specific cortical regions^129,130^, for some of which (e.g., striatum) structural and functional deviations were reported for IDDs^63^. However, although the span task in the current study involves articulation, its relation to motor sequence learning is not straightforward as the latter might be specific to limb movement control^131^.

### Challenging the generally accepted notion of reduced STM in dyslexia

Though word and non-word spans are routinely assessed in dyslexia studies as a measure for STM capacity, most studies of dyslexia have never considered the impact of the frequency of items used in these tasks. The standard Digit Span task, for example, uses very frequent items–digit words, and using such frequent items might especially impair the performance of IDDs, beyond a general STM deficit, if such exists. Thus, the numerous reports of reduced STM in dyslexia are based on the Digit Span (e.g.,^8–10,14,17,89,94^) may in fact reflect a deficit with the utilization of item frequency and not STM per-se. This suggestion gains support from studies showing that the variance in individual reading abilities that is accounted for by STM, can be captured by phonological awareness individual scores (e.g.,^132,133^). Indeed, if a deficit in benefiting from item frequency explains both phonological awareness and STM performance, their contribution to explaining variance in reading might be substantially overlapping.

When words were used, lexical or semantic effects that were not controlled for may have affected performance. For example, such confounds may provide potential explanation for the lack of a reduced word-frequency effect in IDDs^21,22,134–137^. When syllables were used, their frequency was not addressed^21,22,136,137^.

Recently, Kimel et al.^13^ asked about the effect of syllable frequency on spans in dyslexia. They administered a Syllable Span and found that IDDs’ scores benefited less from syllabic frequency. This seems at odds with an earlier study by Rispens et al.^138^, who asked a related question. They administered a non-word repetition task and found that children with dyslexia had greater relative difficulties with items with low vs. high phonotactic probabilities (PP). We propose that this inter-study difference may stem from phonotactics not being equivalent to syllabic frequency since they used bi-phones that disregarded syllable boundaries and might have introduced a confound of syllable frequency in the “low-PP” and “high-PP” conditions. This is especially important since syllables are considered more natural units of processing than single phonemes or sequences of phonemes across word boundaries (e.g.,^70^). For example, in a study that used a masked priming paradigm, a string of letters was presented before a word or a non-word. When this string formed the first syllable of the target word, naming time was reduced compared with a condition in which the string contained an extra or missing letter from the first syllable of the target^139^. Age may also have played a role in this inter-study difference. In the study of Rispens and colleagues, the performance of typically developing children was similar to that of older children with dyslexia. This suggests that children with dyslexia benefit less from exposure, rendering their performance similar to that of less experienced (younger) individuals. Similar findings of a reduced benefit from exposure emerges when sensitivity to language sound repetition is assessed in children with dyslexia^84^, when neural response to phoneme combinations is assessed in children^86^ and adults^140^ with dyslexia, when benefit of syllable frequency in adult IDDs are compared to that of non-native speakers^13^, and when sensitivity to word structure is tested^12,47^.

To summarize, we suggest that, possibly, the numerous reports of reduced STM capacity in dyslexia largely reflect the choice of frequent items (e.g. frequent syllables, digit words in native language) for these tasks, as this choice specifically hampers IDDs’ performance due to their poor utilization of long-term item frequency.

## Conclusion

We measured the benefit from series repetition and the benefit from items’ long-term frequency using the Hebb-repetition task in adults with and without dyslexia. We found no deficit in IDDs’ learning of repeated series. Thus, if there is a serial-order learning deficit in dyslexia, it is probably mild or is not a core deficit in dyslexia—perhaps manifested in some individuals but not in others. However, controls had a higher benefit from item frequency, suggesting that IDDs’ accumulative long-term benefit is reduced.

This reduction might account to a large extent for the deficit reported for IDDs in STM tasks since frequent items (e.g., native language digits) are typically used in these tasks, potentially increasing the measured group difference. Furthermore, these results imply a dissociation between the mechanisms that underlie item frequency and those that underlie repeated sequence learning. We suggest that exposure-independent properties of the items allow better concatenation and lead to higher benefit of sequence repetition, whereas item frequency enhances overall performance but is not specific to sequence repetition.

## Method

The Hebrew University's ethics' Review Board, named *The Institutional Committee for the Use of Human Subjects in Research* specifically approved this study. All procedures were performed in accordance with the relevant guidelines and regulations. A written informed consent was obtained from all participants.

### Assessment battery

Cognitive assessments that were conducted during the first, introductory, session at the lab:

1. The Block Design task (a subtest from the Hebrew version of the Wechsler Adult Intelligence Scale, WAIS-III^141^) was used as a measure of non-verbal intelligence. This task is often used to match groups for non-verbal reasoning.
2. The Digit Span task^141^, a standard STM measure.

Reading and phonological measures:

1. Single word reading: words, pseudo-words and non-words. The pseudo-words were constructed based on Hebrew morphological patterns^142^, and the non-words did not follow Hebrew morphology^31^.
2. Paragraph reading^143^.
3. Phonological awareness: 20 word pairs were orally presented^89,143^. For each pair, participants were asked to swap the initial phonemes of the two words (the "spoonerism" task).

### Participants

We recruited 37 adult participants with dyslexia and 35 age-matched controls via flyers posted around Hebrew University campuses and at two other colleges in Jerusalem. Participants were paid for their participation. They arrived to an introductory session at the lab, which included the tasks in the *assessment battery described above*. Some of the participants also took part in other studies at the lab (e.g.,^12,13^). All participants received all their schooling in Israel. Hebrew was all participants’ mother tongue, and most of them had been born in Israel (the remainder were brought to Israel as very young children, before elementary school).

For the dyslexia group, only participants who also reported early difficulties in reading acquisition (i.e., not only recent difficulties) were invited. Assignment to groups was based on participants’ self-reports and previous formal diagnosis for IDDs and exclusion criteria. For both groups, exclusion criteria included psychiatric medications (other than those for attention deficit^31^), hearing problems, extensive musical background, and below-average cognitive scores (for details see^12^). Participants who reported having dyslexia but did not have any errors in decoding (i.e., perfect non-word reading) were also excluded from the study. Based on these exclusion criteria, one control participant and two participants with dyslexia were not invited to the lab beyond the initial assessment session. Additionally, one control participant’s data were excluded prior to data analysis in order to improve age matching between the groups. This process resulted in 35 IDDs and 33 control participants matched for age and general reasoning skills.

Twenty-five IDDs and 33 controls participated in the Hebb repetition-learning experiment (other participants from the initial pool did not perform the Hebb experiment, but only the introductory session or other experiment at the lab). Three control participants were removed from the analysis: one due to very poor performance in the Consonant–Vowel (open) syllables (CV) part (more than 4 SDs below the group mean) and two in order to match age with the IDD group, resulting in 25 IDDs and 30 controls (Table 2).

**Table 2:** Participant characteristics: general, reading, and phonological abilities.

Measure	Controls	IDDs	Group difference (t; p)
	N = 30 (17 F)	N = 25 (16 F)	
Age (years)	25.3 (2.4)	24.0 (2.8)	1.9; 0.067
Block design (scaled)	12.6 (2.9)	13.4 (2.6)	− 1.0; 0.301
Digit Span (scaled)	11.1 (2.7)	8.0 (2.1)	4.5; 4 × 10^–5^
**Reading accuracy (% correct)**			
Single words	96.8 (4.1)	86.7 (8.2)	5.8; 3 × 10^–7^
Single pseudo-words	89.9 (11.0)	59.0 (18.1)	7.6; 4 × 10^–10^
Single non-words	87.2 (13.1)	49.5 (23.1)	7.4; 4 × 10^–10^
Paragraph reading	98.6 (1.4)	95.1 (4.7)	3.9; 3 × 10^–4^
**Reading rate (words/minute)**			
Single words	95.3 (28.8)	67.0 (25.5)	3.7; 4 × 10^–4^
Single pseudo-words	57.4 (23.7)	31.9 (12.0)	4.8; 10^–5^
Single non-words	41.1 (14.6)	25.7 (8.2)	4.6; 3 × 10^–5^
Paragraph reading	138.3 (21.2)	98.9 (24.6)	6.3; 7 × 10^–8^
**Phonological awareness: spoonerism**			
Accuracy (% correct)	91.3 (6.7)	79.8 (16.5)	3.4; 10^–3^
Rate (items/minute)	9.6 (2.7)	6.2 (3.2)	4.2; 10^–4^

Means and SDs. *F* Female participants.

Participants (IDDs, n = 8) who were taking medication for attention-deficit and/or hyperactivity disorder (e.g., Concerta) did not take that medication on the day they participated in the experiment and performed the initial assessment battery^31^. This subgroup did not significantly differ from the other dyslexia-group participants in age, block design, or the measures of the Hebb-learning experiment (means and slopes).

### Choice of frequent and infrequent syllables

We chose to use Consonant–Vowel (CV) syllables as our frequent items and Vowel-Consonant (VC) syllables as our infrequent items. CV syllables are part of the syllabic vocabulary in almost all known languages^144^, and constitute the majority of syllables in Hebrew^145^. Contrary to CV syllables, VC syllables are rare in languages in general^146^ and particularly so in Hebrew^145^. Converging evidence indicates that in Hebrew, CV syllables are an order of magnitude more frequent than are VC syllables^145^ (discussed in^147^). We verified this assumption by calculating the distribution of syllables in the largest publicly available punctuated corpus of spoken Hebrew (~ 6500 word tokens; The Corpus of Spoken Israeli Hebrew—CoSIH; percent of occurrence in the corpus—mean (SD) for CV syllables: 0.17 (0.17) and for VC syllables: 0.02 (0.03); *t* = 2.76, *p* = 0.014; Table S1 in the supplementary material^13^). Choosing these two syllable types: a very frequent type and an infrequent type, was important in order to verify that the difference in exposure (i.e., familiarity) between the two syllable types is substantial (left vs. right on Fig. 1).

### Procedure

We administered the Hebb repetition-learning task^49^, presenting sequences of nine syllables to the participants in each recall trial and using the same syllables for all sequences^52^. The protocol was administered twice—once with CV syllables and once with VC syllables—in a counterbalanced order across participants. The syllables were digitally recorded by a female speaker, and were presented to the participants through headphones. In each part we administered 30 sequences: 20 non-repeated sequences (“filler sequences”), and 10 repetitions of one sequence (the “Hebb sequence”). The repeated sequence was presented in every third trial, and its position (first/second/third) with respect to the beginning of the paradigm was randomized across participants. The played sequences were prepared in advance by randomizing sequences of syllables so it is unlikely that a frequent bi- or tri-syllabic sequence was presented consistently across participants (35 different sets of 20 non-repeated sequences and one repeated sequence were used).

**Figure 5:**
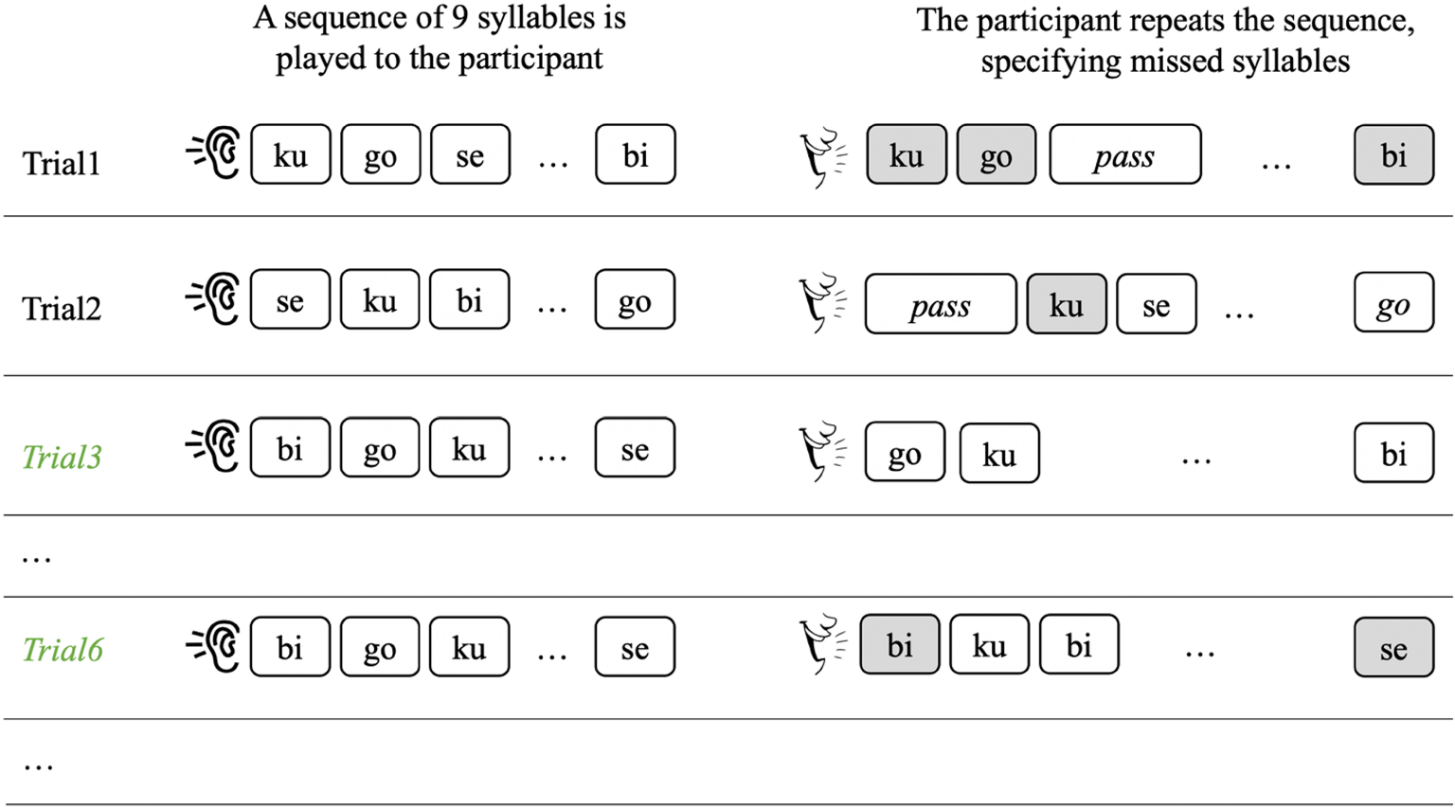
The Hebb-learning experiment procedure. Every third sequence is repeated (marked in italic green). In order to be scored as “correct”, a syllable must be recalled in the correct position (marked in grey).

The paradigm was as follows: in each trial a prerecorded sequence was presented, and the participant was asked to orally reproduce the sequence, paying special attention to the order. If a participant knew an item was missing from the recall, the participant was asked to verbally indicate it (e.g., *“fe lu pass bi…*”; Fig. 5). The score of each sequence was the fraction of syllables that were correctly reproduced with respect to their position (e.g., if a participant recalled two syllables in their correct position, the score for that sequence would be 2/9 ~  = 0.22). Participants received points for correct relative ordering of syllables. For example, if the response to the sequence *“zi go fe sho lu se ri ku bi”* was *“go ku pass pass lu se and then sho bi at the end”,* the participant received a score of 3/9 for the syllables *lu, se, bi*.

In order to study the dynamics of learning the repeated sequence (Hebb-learning), two regression lines were fitted to each participant’s scores across trials: one for the repeated sequence and one for the non-repeated sequences. In each triplet of sequences, there was one sequence that repeated every third trial (e.g., trials number 1, 4, 7, … 28), resulting in 10 data-points for the regression line for the repeated condition. The scores for the two non-repeated sequences in a triplet were averaged (e.g., trials 2 and 3, 5 and 6, … 29 and 30) and this value was treated as one data point for the regression, resulting in 10 data-points the non-repeated condition. The difference in the slopes of these two regression lines was used as a measure of serial-order learning (after^52–54^). Given the reduced reliability of slope effects^51^, we also assessed *Repetition*, *Syllable Type* and *Group* effects (and their interactions) using mean scores of each group under each condition: CV and VC for repeated and non-repeated sequences.

### Statistical analysis

The main analysis of means and slopes was done using a mixed-design ANOVA. In questions for which it was important to show that there was no group difference, we used the Bayesian approach, and reported a Bayes factor. Bayes factor compares the probability of the observed data (i.e., results of the experiment) given the null hypothesis ("the groups do not differ") against the probability of the observed data given the alternative hypothesis ("the groups differ"), thus allowing for a comparison of the two hypotheses. Therefore, unlike traditional frequentist statistics, it can provide support for the null hypothesis rather than merely accepting or rejecting the alternative. We used a calculator kindly provided by the *Perception and Cognition Lab*^76^. It is generally accepted that Bayes factor (B_01_) values that are greater than 3 are considered to support the null (H_0_) hypothesis (3–10 is some support, 10–30 is strong support).

Recently, a methodological paper questioned the Hebb-learning task reliability for assessing repeated series learning ^51^. Bogaerts and colleagues have studied the Hebb-learning paradigm systematically, within and across a single experiment, two separate sessions, and the items used in the task. They found that while the individual performance for the filler sequence (without repetition) is highly stable, the Hebb-learning effect is not. Following this, we have adopted a similar approach, and tested the correlation of Hebb learning between the two runs (CV and VC) in order to verify the reliability of the Hebb-learning results in our study.

## Supplementary Information

Supplementary Table S1.
