# Supplementary material for: Repeated series learning revisited with a novel prediction on the reduced effect of item frequency in dyslexia
Source: Sci Rep. 2022 Aug 8;12:13521. doi: 10.1038/s41598-022-16805-z (PMC9359986; doi:10.1038/s41598-022-16805-z)
Supplement: Supplementary file 1 — Supplementary Table S1. [file 41598_2022_16805_MOESM1_ESM.pdf]

Table S1

**Consonant-Vowel (CV) syllable**	**Frequency (% out of 16,940)**	**Vowel-Consonant (VC) syllable**	**Frequency (% out of 16,940)**
zi	0.05	iz	0.00
go	0.06	og	0.00
fe	0.02	ef	0.05
sho	0.18	osh	0.00
lu	0.52	ul	0.00
se	0.22	es	0.05
ri	0.34	ir	0.06
ku	0.06	uk	0.00
bi	0.10	ib	0.00

Frequency (in percent) calculated from the occurrence counts of the syllables used in the study. The CV syllables are significantly more frequent than the corresponding VC syllables (mean (SD) for CV syllables: .17 (.17) and for VC syllables .02 (.03); $t = 2.76$, $p = .014$).
